# Investigation of key chemical species from durian peduncles and their correlations with durian maturity

**DOI:** 10.1038/s41598-021-92492-6

**Published:** 2021-06-25

**Authors:** Preeyarad Charoensumran, Kornkanya Pratumyot, Tirayut Vilaivan, Thanit Praneenararat

**Affiliations:** 1 Department of Chemistry, Faculty of Science, Chulalongkorn University, Phayathai Rd., Pathumwan, Bangkok, 10330 Thailand; 2 The Chemical Approaches for Food Applications Research Group, Faculty of Science, Chulalongkorn University, Phayathai Rd., Pathumwan, Bangkok, 10330 Thailand; 3 Organic Synthesis, Electrochemistry and Natural Product Research Unit, Department of Chemistry, Faculty of Science, King Mongkut’s University of Technology Thonburi, Bangkok, 10140 Thailand; 4 Organic Synthesis Research Unit, Department of Chemistry, Faculty of Science, Chulalongkorn University, Phayathai Rd., Pathumwan, Bangkok, 10330 Thailand

**Keywords:** Mass spectrometry, NMR spectroscopy, Sustainability, Peptides, Secondary metabolism

## Abstract

The popularity and high price of durian make quality control in terms of ripeness very important, which in turn depends heavily on harvesting at an appropriate maturity stage. To date, reports on data-driven methods for maturity prediction are scarce, with many rather focusing on ripeness prediction. Herein, we report the first disclosure of key molecular markers in the liquid extract of durian peduncle that can be a predictive tool for maturity. Multiple chromatographic and spectroscopic techniques including TLC, HPLC, PS-MS, LC–MS/MS, and NMR, were used to characterize chemical profiles of the aqueous extracts from peduncles at different ages. Four compounds that show positive correlations with maturity were identified as sucrose, asparagine, arginine, and pipecolic acid, with asparagine as the most abundant species. This finding paves the way for more research of high impact such as the relationship between biochemical reactions in peduncle and pulp, and the development of accurate and non-destructive sensors for maturity prediction.

## Introduction

Durian (*Durio zibethinus*) is widely known as “the King of Fruits” in Southeast Asia due to its unique taste and aroma. The most popular cultivar of durians grown commercially in Thailand is “Monthong”, which is characterized by fleshy light yellow pulp and mild aroma^1,2^. In the past few decades, Thai durians have gained increasing popularity, with major exports to China, Taiwan, and Hong Kong^3^. Recently, Thailand ranked in number one as the top exporter of fresh durians (1.4 billion USD in 2019)^4^, clearly highlighting its status as one of the most economically important fruits in Thailand. Accordingly, the quality of durians in various aspects plays a critical role in maintaining sustainable export activities.

Among these, the maturity of durians is a critical factor that growers need to pay attention to. That is, high-quality durians should be harvested at an appropriate maturity stage. If the durian fruits were cut from the tree sooner than this optimal range, they will not be able to efficiently ripen. Considering the high price of durian (estimated retail price at 6–12 USD/kg of a 3–5 kg size fruit), such unnecessary loss should be kept minimum. Nevertheless, accurate estimation of appropriate maturity for harvesting is non-trivial, typically requiring multiple pieces of information brought into consideration. One prominent factor is the number of days after anthesis (DAA), which range from 90 to 150 days depending on the cultivar^5^. While seemingly objective and straightforward, the day-counting can be highly tedious and impractical due to the fact that fruits can develop at different times, even for those from the same tree. Other factors include acoustic tapping and the observation of physical appearance of the fruit^5^. These conventional techniques are subjective by nature and depend heavily on the harvester’s experience, thus rendering them inconsistent and unreliable. Therefore, various research groups had proposed systematic and unbiased scientific method regarding with the maturity and ripeness of durians. Interestingly, except in a handful of cases^6–9^, most of these studies focused on ripeness^10–13^, which is the process that occur (or may not occur) after harvesting. Thus, these studies do not directly help improve the decision making regarding a suitable harvesting time.

**Figure 1:**
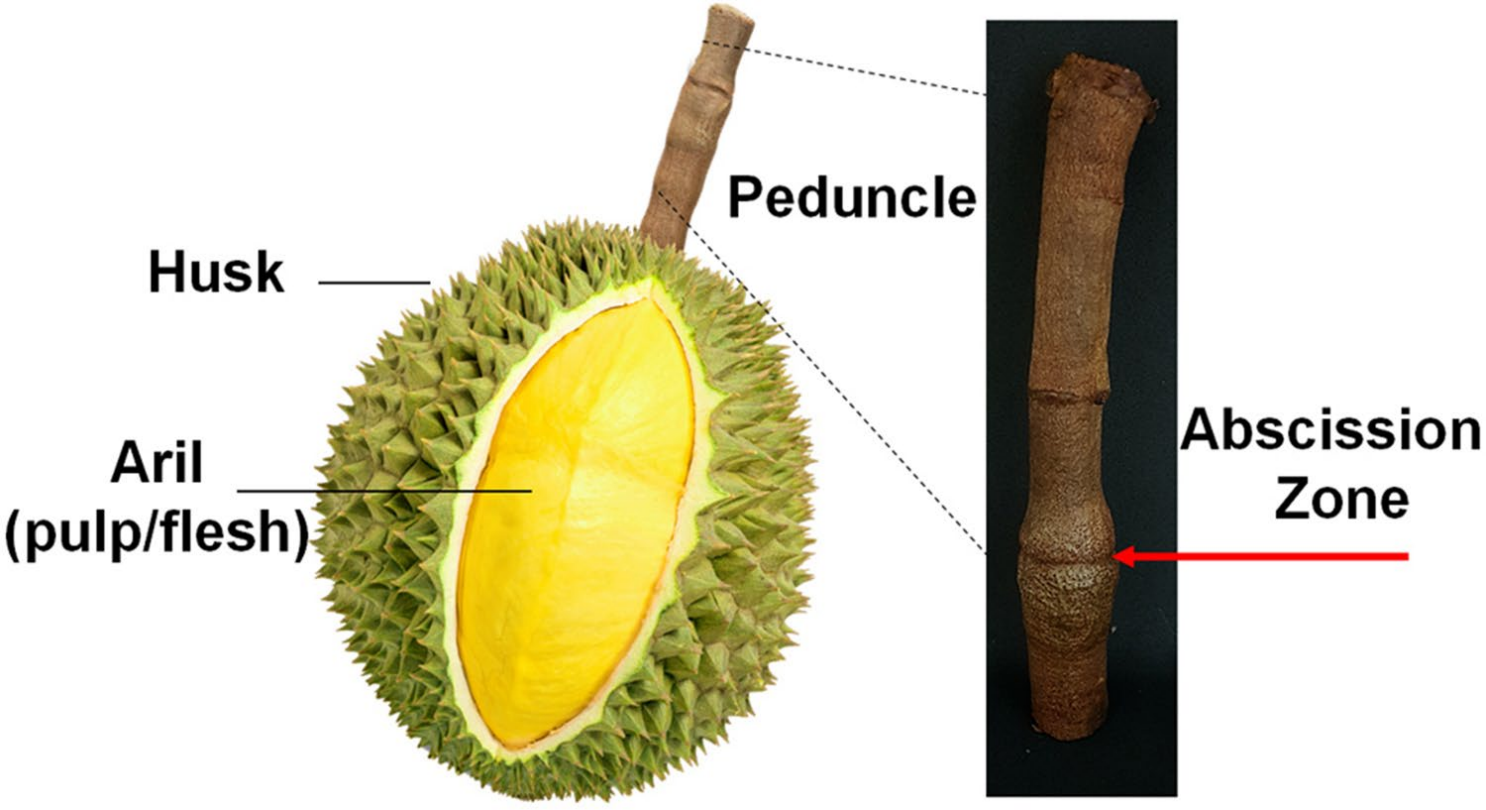
Illustrations of parts of durian fruit and the peduncle of durian (inset).

In this study, we systematically studied the liquid extracts from the fruit’s peduncle (Fig. 1) and uncovered some key components that are indicative of durian’s maturity. While some studies on durian peduncles to correlate with the maturity exists^7,9^, they were based on physical methods and molecular information was entirely lacking. Herein, we combined multiple chemical techniques to analyze the chemical contents of the liquid extracts from durian fruits peduncle, along with exploring an alternative option, *i.e.,* paper-spray mass spectrometry (PS-MS) to obtain the analysis results more rapidly. Interestingly, apart from the presence of some sugars, additional compounds were also detected, and these compounds showed a clear relationship with the maturity of the durian fruits. This finding may be useful in further developments of a new predictive tool for the maturity of durians.

## Materials and methods

### Materials

All chemical reagents and solvents were purchased from Sigma-Aldrich, Merck, TCI Chemicals, and RCI Labscan. Sucrose-*d*_2_ (β-D-[6,6'-^2^H_2_]fructofuranosyl-α-D-glucopyranoside) was purchased from Omicron Biochemicals. The papers used in this study are Whatman 1Chr chromatography papers. Each “Monthong” durian fruit was collected (with permission from the grower) from different trees of approximately 20 years old that were grown in an orchard at Rayong province, Thailand (geographic coordinates in decimal degrees: 12.695648717424858, 101.35059404973144).

### Sample preparation

It is noted that all experiments were performed in accordance with relevant guidelines and regulations. To study the optimal harvesting maturity of durian, whole “Monthong” durian fruits (three for each maturity) were collected at 13 weeks, 15 weeks and 17 weeks after fruit set. All collected fruits were transported to the laboratory within one full day. Thereafter, the peduncle was immediately cut off from each fruit and kept at – 20 °C until the analysis was performed (usually in less than three days). A portion of frozen stem (1.5-cm long sections above the abscission zone, which weighed around 3.4–5 g) after thawing was chopped into small pieces (about 0.5 × 0.3 × 0.2 cm), mixed with Milli-Q water (at a ratio of 0.5 g/mL water), and the resulting suspension was sonicated for 5 min, followed by incubation at room temperature (25 °C) for 1 h. The extracted solution was directly used for the PS-MS analysis without any further treatments. For high performance liquid chromatography (HPLC) analysis, a 500-μL aliquot was mixed with an equal volume of MilliQ water, followed by filtration through a 0.2-μm syringe filter. The resulting solution was then subjected to HPLC analysis (see the HPLC conditions in “High performance liquid chromatography (HPLC) analysis” section).

For the analysis of the durian pulps, a sample of 1-g blended pulp was mixed with 10 mL of 50% (v/v) aqueous ethanol. The mixture was then heated at 82 °C for 25 min in a heating block^14^. The solution was then filtered by a 0.2-μm syringe filter for HPLC analysis (see the HPLC conditions in “High performance liquid chromatography (HPLC) analysis” section).

### Thin layer chromatography (TLC) for sugar analysis

Standard sugar samples (glucose, fructose, and sucrose) were dissolved in water at 1 mg/mL and used as standards for comparison with real samples. Both standard solutions and samples were repeatedly spotted (1 μL × 5) at the predefined positions onto a silica gel GF254 TLC plate. The TLC plate was developed in a mixture of chloroform, acetic acid, and water (3:3.5:0.5 v/v) as the mobile phase. The development was performed twice for the same TLC plate in the same mobile phase. After being air-dried, the developed TLC plate was sprayed with a staining reagent for sugars (a freshly prepared mixture of 1 g of diphenylamine and 1 mL of aniline in 100 mL acetone, and 10 mL of 85% orthophosphoric acid)^15^ to visualize the spots.

### High performance liquid chromatography (HPLC) analysis

All HPLC experiments with a refractive index detector (RID) were performed with the following parameters: Model: Shimadzu Co-Sense Series (Kyoto, Japan), column: NH_2_ Analytical HPLC Column 4.6 × 250 mm, 5 μm, injection volume: 20 μL, mobile phases (isocratic): 80% acetonitrile in water, flow rate: 1.5 mL/min. For sugar quantification and calibration, a standard solution mixture of D-fructose, D-glucose, and D-sucrose were prepared with two different solvent systems (water and 1:1 ethanol:water) at five different concentrations (0.5, 1.0, 5.0, 7.5, and 10.0 mg/mL). The HPLC measurements were completed in six replicates. Limit of detection (LOD) and limit of quantification (LOQ) were calculated from 3.3*S*_b_/*m* and 10*S*_b_/*m*, where *S*_b_ is the standard deviation of regression line and *m* is the slope of the calibration plot.

### Paper spray mass spectrometry (PS-MS) analysis

In the PS-MS analysis, the filter paper was cut into isosceles triangular shape with a dimension of 0.6 cm (base) × 1.2 cm (height) and then washed by shaking with methanol for 10 min and rinsed with methanol. The cut pieces of paper were air-dried under ambient condition. Each sample (standard or real samples) was prepared by diluting an aliquot of 100-μL sample (or standard solution) in water with another aliquot of 100-μL methanolic solution of sucrose-*d*_2_ (the internal standard). Five μL of the resulting 1:1 methanol:H_2_O solution was then loaded onto the cut paper piece, and allowed to dry at atmospheric pressure. The PS-MS analysis was performed in positive ionization mode by the addition of 10 μL of methanol with 0.1% formic acid as the spray solvent. All PS-MS experiments were performed on a Thermo Scientific TSQ Quantum EMR triple quadrupole MS. Mass spectra were acquired over the *m/z* 50–500 range. Spraying voltage was set at 3.5 kV by using a DC power supply (3B scientific model U33010). Selected reaction monitoring (SRM) was used for all quantification experiments with the following parameters: capillary temperature = 300 °C; scan width = 1.000; scan time = 0.500 s; Q1 peak width = 0.70; Q3 peak width = 0.50; collision cell (Q2) Ar pressure = 1.5 mTorr. To quantify the sucrose concentration, the [M + Na]^+^ peak of sucrose (*m/z* 365) was used as the parent ion (Q1), which provides *m/z* 203 as the daughter ion (Q3) (collision energy = 33 V; tube lens voltage = 102 V). Sucrose-*d*_2_ was used as the internal standard with Q1 as *m/z* 367 and Q3 as *m/z* 205. For calibration plots, five sucrose concentrations were prepared (0.1, 0.5, 1.0, 1.5, and 2.0 mg/mL in water) and spiked with an equal volume of methanol solution of sucrose-*d*_2_ (final concentration of 1.25 mg/mL). Each measurement was performed in seven replicates.

### Liquid chromatography–mass spectrometry (LC–MS) analysis with quadrupole time-of-flight (QTOF) MS

An Exion HPLC coupled with QTOF X500R MS system (SCIEX, USA) was used in this experiment with the following parameters.

HPLC conditions: ZORBAX RRHD Eclipse-C18 column (2.1 × 50 mm, 1.8 µm) (Agilent, USA); mobile phases (gradient) were 0.1% formic acid in water (solvent A), and acetonitrile (solvent B). Time programs (at a flow rate of 0.5 mL/min and column temperature of 30 °C) were 0–5.0 min for 5–20%B, 5.0–6.0 min for 20–50%B, 6.0–7.0 min for 50–100%B, 7.0–8.0 min for 100%B, 8.0–9.0 min for 100–5%B, 9.0–10.0 min for 5%B). The injection volume was 5 μL.

Mass spectrometry (ESI as an ionization source): spray voltage = 5500 V, declustering potential = 50 V, temperature = 500 °C, ion source gas 1 and 2 = 45 psi, curtain gas = 30 psi, collision energy = 10 V, TOF mass range = 100–1000 Da.

### Nuclear magnetic resonance (NMR) spectroscopy experiment

The liquid extract was first purified by flash column chromatography in hydrophilic interaction (HILIC) mode with the following parameters. A Puriflash 450 (Interchim) system with a Silica HP-50-F0012 (14 g, 2 cm × 8 cm, 50 μm) flash cartridge (flow rate = 15 mL/min) was used. The mobile phases (gradient) were acetonitrile (solvent A), and water (solvent B). Time programs were 0–3 column volume (CV) for 10–20%B, 3–5 CV for 20–30%B, 5–7 CV for 30–40%B, 7–10 CV for 40%B. All fractions were collected by an automated fractions collector and analyzed by TLC. The fractions showing an R_*f*_ value of 0.36 (8:2 acetonitrile:water, purple spot with *p-*anisaldehyde staining reagent) according to TLC analysis were combined and the solvents removed by rotary evaporation at 45 °C (using acetonitrile as a co-solvent). The residue was dissolved in D_2_O and then analyzed by ^1^H NMR (256 scans; JEOL JNM-ECZ500R/S1 operating at 500 MHz for ^﻿1^H).

### Liquid chromatography–mass spectrometry (LC–MS) analysis with ion-trap triple quadrupole (QTRAP) MS

A 1290 Infinity II HPLC (Agilent, USA) coupled with QTRAP 4500 MS system (SCIEX, USA) was used in this experiment with the following parameters.

HPLC conditions: ZORBAX RRHD Eclipse-C18 column (2.1 × 50 mm, 1.8 µm) (Agilent, USA); mobile phases (gradient) were 0.1% formic acid in water (solvent A), and acetonitrile (solvent B). Time programs (at a flow rate of 0.5 mL/min and column temperature of 30 °C) were 0–1.0 min for 2%B, 1.0–4.0 min for 2–80%B, 4.0–6.0 min for 80%B, 6.0–6.1 min for 80–2%B, 6.1–8.0 min for 2%B). The injection volume was 1 μL.

Mass spectrometry (ESI as an ionization source) was performed as following parameter; spray voltage = 5500 V, temperature = 500 °C, ion source gas 1 and 2 = 18.0 and 0.0 psi, curtain gas = 8.0 psi. Multiple reaction monitoring (MRM) was used to quantify sucrose, asparagine, arginine and pipecolic acid simultaneously. The optimized parameters of each compounds are presented in Tables S1. Calibration curves for all analyzed species were obtained in the range of 100–1000 ng/mL, except asparagine (100–5000 ng/mL). All curves were obtained with good linearity (R^2^ = 0.9989–0.9996, Figure S1). Each measurement was performed in six replicates.

## Results and discussion

### Identification and content determinations of sugars in durian fruit peduncles

In this study, durians were collected from 13 (13 W), 15 (15 W), and 17 weeks (17 W) after fruit set. It should be noted that 17-week timepoint (~ 120 days) is generally viewed as an approximately optimal maturity for the Monthong cultivar. Durian fruits harvested earlier than this will not ripen, while harvesting later than this optimal timepoint can substantially reduce its shelf life.

**Figure 2:**
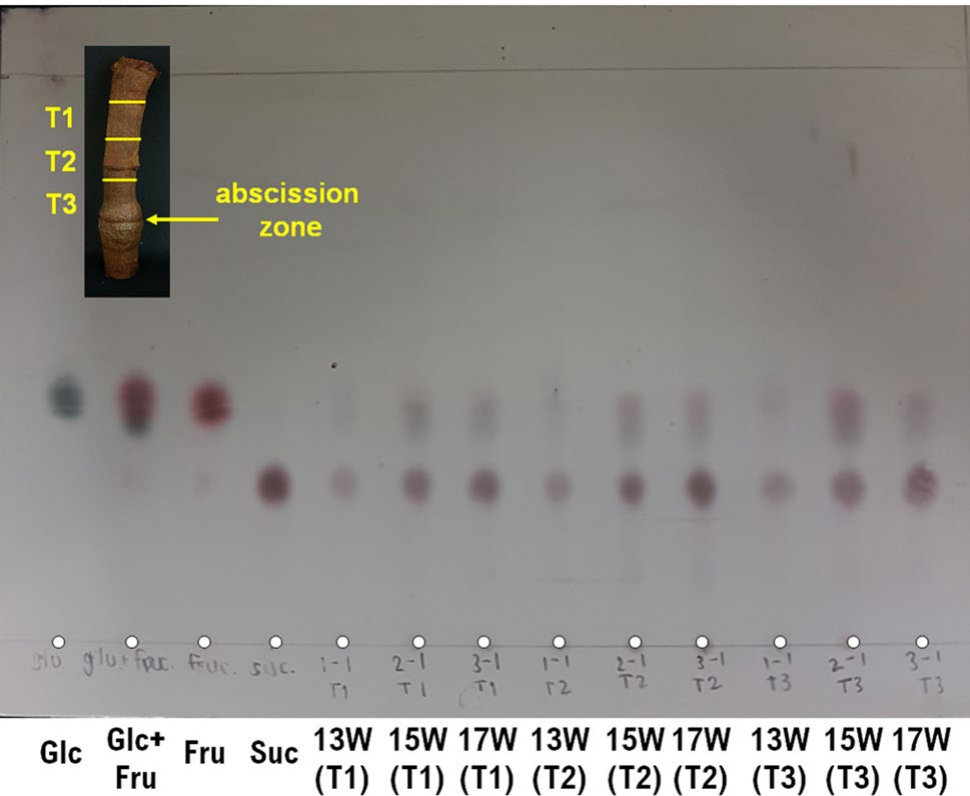
Thin layer chromatogram (TLC) of standard sugar solutions (glucose, glucose and fructose, fructose, and sucrose respectively), and liquid extracts from the peduncle of 13 W, 15 W and 17 W (with T1, T2 and T3 as different portions measured from the abscission zone).

According to local wisdoms, tasting the sweetness of the liquid extracts from the peduncle (Fig. 1) as a measurement of sufficient maturity of durians has been used by some durian growers in Thailand. Therefore, analyzing the amounts and types of sugars within this liquid is the most intuitive means to start with. A small sample from the peduncle part immediately above the abscission zone was taken, chopped into small pieces, and then extracted with water for further analysis. For a preliminary investigation, thin layer chromatography (TLC) was employed to analyze the type and sugar contents in the extracted liquids. As shown in Fig. 2, all samples at different ages contained all three sugars, namely glucose, fructose, and sucrose. Although glucose and fructose possessed similar *R*_*f*_ values, they were stained to give different colors. While the intensity of the colored spots may not be entirely quantitative, estimating the intensities of spots within the same TLC experiment can be used to compare the amounts of respective compounds. Based on this assumption, it can be roughly estimated that the amount of sucrose increased with increasing maturity. This is in line with previous studies where sucrose was also found to be the main sugar component in durian pulps^11,16,17^. On the other hand, the amounts of both glucose and fructose were generally low at 13 weeks and increased at 15 and 17 weeks. We have also examined whether the distance from the abscission zone affects the analysis. This was done by sampling the peduncle above the abscission zone into three parts, *i.e.,* T1, T2, and T3 (1.5 cm apart, Fig. 2 inset). As shown in Fig. 2, this variation seemed not to have much effect on the content of sugars at the same maturity. Therefore, this variable was not further studied in the subsequent experiments. In brief, TLC analysis suggested that the amounts of sugars, especially sucrose, indeed varied with different maturity stages. Therefore, more quantitative analyses were followed to give a better understanding.

**Figure 3:**
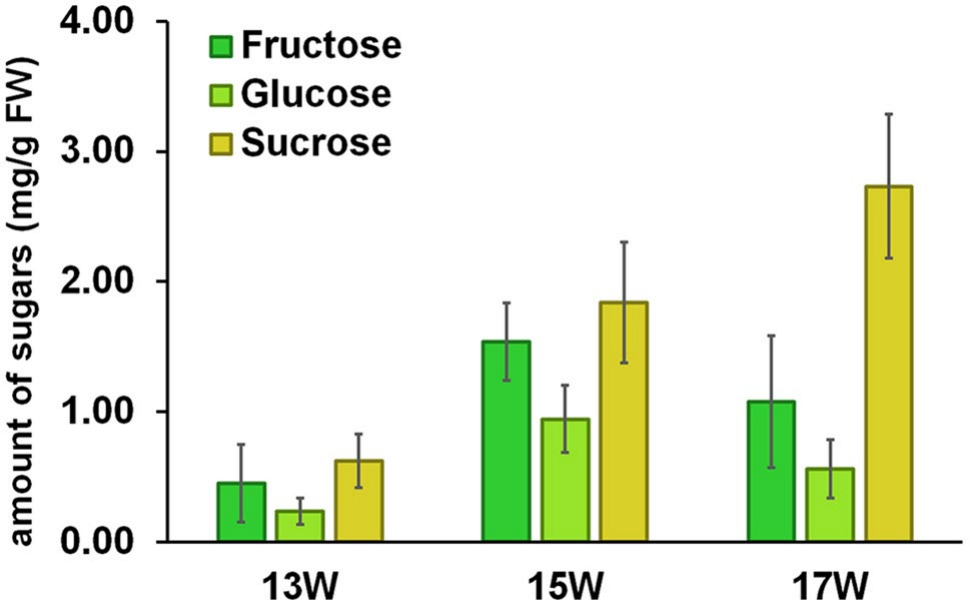
The amounts of three sugars, namely fructose, glucose and sucrose, in the peduncle of durian at different maturities (13, 15, and 17 weeks) as determined by HPLC-RI.

While sugars are relatively simple molecules, the detection and quantification of sugars in food samples cannot be directly performed on conventional HPLC–UV systems due to the absence of chromophores. Therefore, unless some modifications/derivatizations were done^18,19^, HPLC with refractive index (RI) detector^20,21^, or evaporative light scattering (ELS) detector^22,23^ were typically used. Hence, the sugars contents of the peduncle parts were determined by HPLC coupled with an RI detector. The HPLC analysis results (Fig. 3, numerical data in Table S2) gave similar trends to the TLC analysis. First, sucrose was found to be the most abundant sugar in this extract, and its amount significantly increased with increasing ages (0.62–2.73 mg/g fresh weight (FW)). This is in line with previous studies in durian pulp where sucrose was also the major sugar component^11^. On the other hand, the amounts of glucose and fructose seemed to reach their peaks at 15 weeks and then decreased afterward, a trend of which was partly observed in the aforementioned TLC analysis (Fig. 2). To further relate the content of sugars found in the samples with the traditional tasting from farmers, the sweetness of each sample with different maturities was also calculated (see Table S3 and calculation method in supplementary information)^24^. Since all sugars can contribute to the sweetness that one perceives, tasting as a means to estimate the maturity may be prone to error if the contents of some sugars do not increase with increasing ages of the fruit. Interestingly, the calculation indeed showed that the calculated sweetness values from the 15-week sample and the 17-week sample appeared to be very similar (2.20 vs. 2.23, respectively). This means that one may feel the same sweetness from two fruits with two-week difference in maturity, leading to erroneous estimation. On the other hand, any analytical method that determines only the amount of sucrose is a much better method to estimate the maturity stage of durians.

After confirming the direct relationship of sweetness (or more specifically the amount of sucrose in the liquid extracts from durian peduncles), and the maturity stages, it would be of interest to investigate the relationship between the sugar contents from the peduncle and in the pulp. Figure S2A (numerical data in Table S4) showed that in the case of durian pulp, the amount of sucrose dominated in all maturities, reaching as high as 19.6 mg/g FW at 17 weeks. Interestingly, despite having different magnitudes, the amounts of sucrose in the pulp and in the peduncle showed a fairly linear correlation with each other (Figure S2B), indicating that both show the same trend of having increased amount of sucrose with longer maturity.

### PS-MS as a rapid analysis of sucrose in the peduncle area

**Figure 4:**
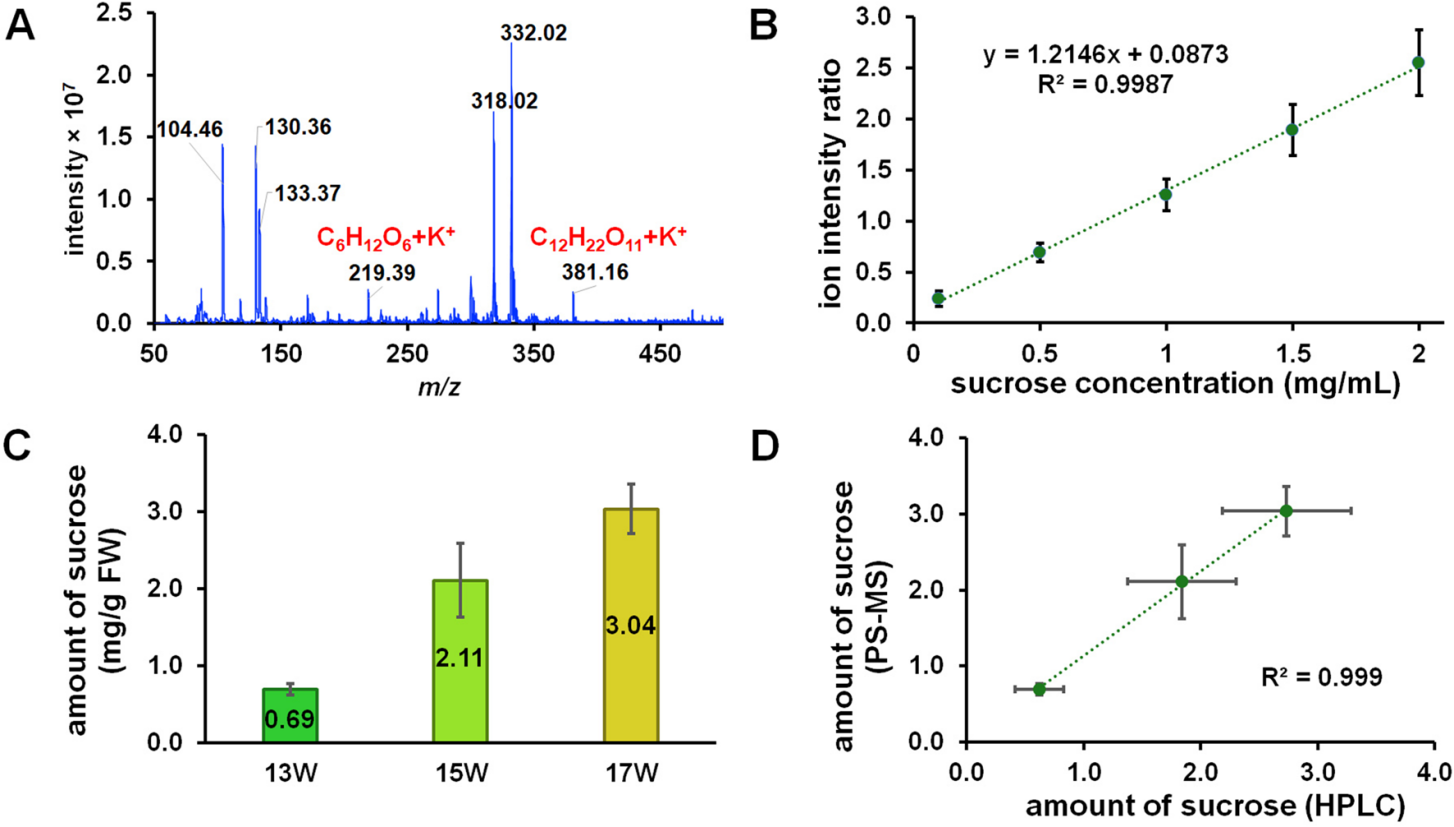
(**A**) Full-scan mass spectrum of liquid extract by PS-MS; (**B**) Calibration plots between the ratio of the intensities of the product ion from sucrose (*m/z* 203) over that from sucrose-*d*_2_ (*m/z* 205); (**C**) the amounts of sucrose from durian peduncles at 3 different maturities analyzed by PS-MS; and (**D**) a scattered plot showing the correlation between determined amount of sucrose from HPLC and PS-MS analyses.

Although HPLC is a standard technique for the determination of sugar contents, some sample preparation for heterogenous samples, *e.g.,* filtration, is necessary to avoid issues with the instrument such as clogging up the lines or HPLC columns. Thus, PS-MS, an ambient ionization technique coupled with mass spectrometry, can offer a solution to the aforementioned limitation due to its unique features including the combination of sample collection, separation, ionization into one operation step^25,26^. Herein, the full-scanned, positive-mode MS spectrum (Fig. 4A) of the peduncle liquid extract with paper spray ionization indicated the presences of monosaccharides, presumably glucose and fructose ([M + K]^+^, *m/z* 219), which are indistinguishable by simple mass spectrometry, and disaccharide, presumably sucrose ([M + K]^+^, *m/z* 381). To explore the aspect of quantification, we focused on the disaccharide peak and employed the selected reaction monitoring (SRM) method to quantify the amount of this *m/z* species through its daughter ion at *m/z* 203. As shown in Fig. 4B, the calibration plot created from the ion ratio of sucrose (*m/z* 203) and sucrose-*d*_2_ (*m/z* 205) as the internal standard showed good linearity. The method exhibited LOD and LOQ at 0.0835 mg/mL and 0.253 mg/mL, respectively, which were sufficient to cover the amount of sucrose at the lower range (13 weeks) of this experiment. It should be noted that the detection limit in this work was 443 times more sensitive than that reported by Collete et al.^27^, presumably due to the use of the SRM in the present study. Thereafter, this method was applied for the determination of sucrose in real samples (Fig. 4C). Gratifyingly, the PS-MS analysis indeed provided a clear trend of increasing sucrose concentrations with increasing maturities (0.69–3.04 mg/g FW). When compared with the result obtained for HPLC or even QTRAP-MS (see below), the data from PS-MS correlate well with these other sets of data, as evident in a scattered plot in Fig. 4D (compared with HPLC). Given that the liquid extract is relatively thick, improper sample preparation may clog HPLC columns easily. Therefore, an alternative method with simple preparation like PS-MS can be a promising tool for the rapid, yet quantitative, determination of sucrose in the durian peduncle extracts, which in turn would be a good indication of the maturity of durians.

### Identification of other chemical species in the liquid extract from durian peduncles

**Figure 5:**
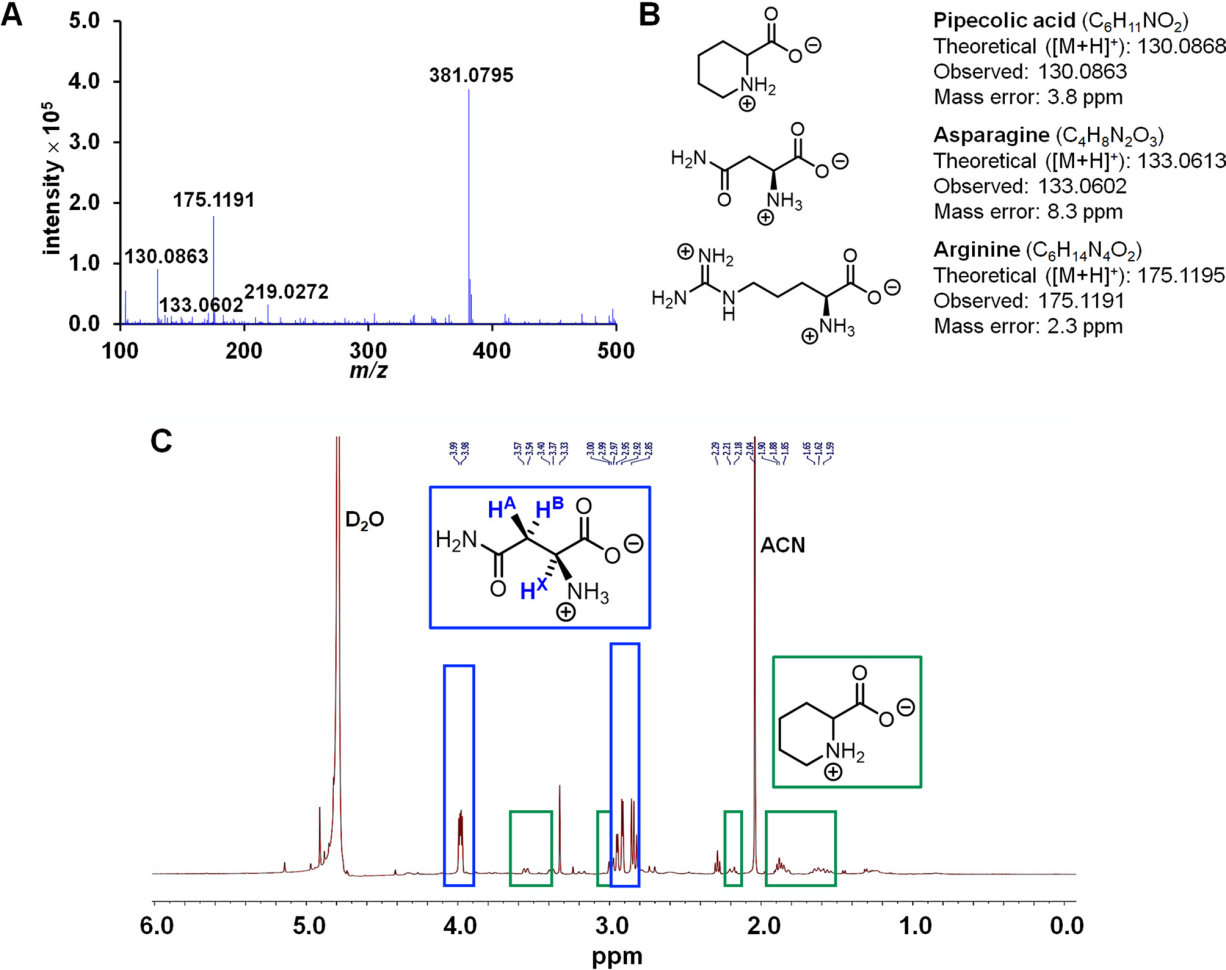
(**A**) MS spectrum of the liquid extract (15 weeks) obtained from LC–MS/MS (QTOF); (**B**) the chemical structures of identified compounds; (**C**) ^1^H NMR (D_2_O, 500 MHz) spectrum of partially purified liquid extract with peaks associated with asparagine in blue boxes, and those with pipecolic acid in green boxes.

While the correlation of sugars in the liquid extract from the durian peduncles and maturity were proven, we were also interested in finding other species that can potentially be used as alternative indicators of sufficient maturity. In fact, the results from PS-MS (Fig. 4A) revealed the presence of some additional species that may increase with the increased maturity of the durian fruits. A preliminary screening in LC–MS/MS (QTRAP) indicated that three additional peaks with the *m/z* values of 130, 133, 175, along with that from sucrose at *m/z* 365, gave relatively high signal intensities. Importantly, these signals show a trend of increasing intensity with increasing ages, hence they are considered as potential maturity markers. In order to allow for accurate quantification, *i.e.,* by creating calibration plots of standard solutions, the identities of these chemical species must be known. Therefore, more accurate molecular characterizations were performed to confirm the identities of these three new peaks. The liquid extract (at 15 weeks) was analyzed by a quadrupole time-of-flight (QTOF) MS to obtain high-resolution mass data (Fig. 5A). As previously seen in the PS-MS experiments, potassium adducts of sugars, *i.e.,* species with *m/z* 219.0272, and *m/z* 381.0795, were found and assigned to be monosaccharides (likely glucose and fructose), and disaccharide (likely sucrose), respectively. In addition, the three aforementioned species were confirmed to have the *m/z* values (exact mass) of 130.0863, 133.0602, and 175.1191. Based on the exact mass values of [M + H]^+^ species, the three peaks were assigned to pipecolic acid, asparagine, and arginine, respectively (Fig. 5B).

To gain even higher confidence in the structural assignment, the ^1^H NMR technique was also used to further confirm the identity of the proposed compounds. After an extensive chromatographic separation in HILIC mode, we managed to partially purify the crude extract that gave some useful information in ^1^H NMR spectroscopy (Fig. 5C). The characteristic ABX pattern of asparagine was clearly identified at 3.97–3.99 (1H, dd) and 2.81–2.95 (2H, dd) ppm. Albeit with smaller intensities, the peaks associated with pipecolic acid can also be assigned by comparing with the NMR data of pure pipecolic acid. These include the signals at 1.59–1.65 (2H, m), 1.85–1.90 (2H, m), 2.18–2.21 (2H, m), 2.97–3.00 (1H, td), 3.37–3.40 (1H, m) and 3.54–3.57 (1H, dd) ppm. Notably, peaks from arginine were not clearly visible in the NMR spectrum. At first glance, this may appear to be contradicting with the QTOF data shown above, where the MS peak of arginine was the most prominent among the three species. Nevertheless, it should be noted that the intensities of MS data cannot be directly compared among compounds of different natures which may affect the ability to ionize. As a highly basic molecule, arginine can be readily ionized by taking up a proton. This in turn leads to exceptionally high signal in MS despite its presence in only small amounts. With calibrated quantification (see above), it was shown that the amount of arginine was indeed the smallest among these species and hence may not be easily detected by NMR.

**Figure 6:**
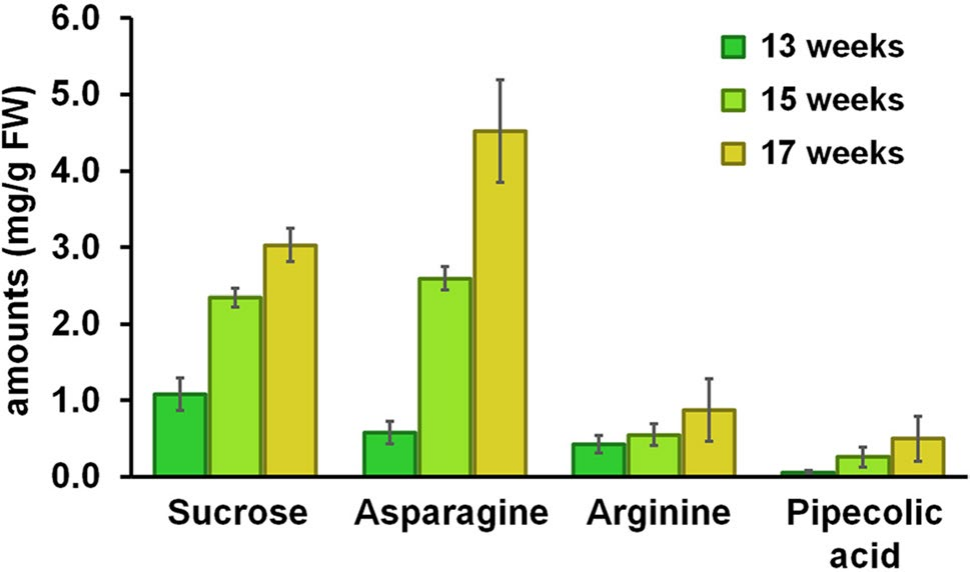
The amounts of sucrose, asparagine, arginine, and pipecolic acid in the peduncle of durian at different maturities (13, 15, and 17 weeks) as determined by LC–MS/MS (QTRAP).

As alluded above, signals from asparagine, arginine, pipecolic acid, and sucrose appeared to show positive correlation with maturities. Hence, to provide real quantification, all peaks were optimized for their suitable daughter ions to be used in the MRM mode (detailed parameters in supplementary information), and quantification of these four ions was subsequently carried out (using calibration plots created from the pure form of all four compounds). As shown in Fig. 6 (numerical data in Table S5), all compounds shared a similar trend of being increased with increasing maturity, with asparagine as the compound with the highest amount at all ages (reaching around 4.5 mg/g FW at 17 weeks maturity). Notably, both asparagine and pipecolic acid were previously reported to be highly abundant metabolites in durian pulps^28^. Hence, there seems to be a relationship between the contents of some chemical species between durian pulp and peduncle, which require further studies for better understanding. In practical senses, this study revealed that, apart from sucrose, asparagine from the peduncle extract can also serve as a potential maturity indicator for durian.

In conclusion, we have performed multiple experiments to scientifically validate the long-known wisdom among durian growers—can one use the sweetness of the liquid extract from durian fruits peduncle as an indication for the maturity stage of durian fruits? First, analyzing the sugar content confirmed that there was indeed a rise in the level of sucrose, which is the major sugar that contributes to sweetness, in the liquid extracts from peduncles along with the maturity of the durian fruit. This was confirmed by multiple techniques including HPLC-RI, LC–MS/MS (QTRAP), and PS-MS. We have demonstrated that the PS-MS technique was a robust tool for sugar monitoring that can be conveniently performed and gave equivalent performance to HPLC. Interestingly, LC–MS/MS analyses of the liquid extracts revealed additional chemical species which were identified as asparagine, arginine, and pipecolic acid—all of which showed the trend of increasing amounts with increasing ages. In particular, the amount of asparagine was found to be higher than that of sucrose in all maturity stages and thus it is a potentially useful new marker for indicating durian maturity. Overall, this study opens a new avenue for further studies in both fundamental and applied aspects. For instance, the relationship of various compounds in the pulp and the peduncle at any maturity stage can be investigated, which may lead to a better understanding of the underlying biochemical process. On the other hand, a chemical sensing system that can specifically detect sucrose and/or asparagine can be fabricated and directly used to more accurately gauge the maturity of durians in a non-destructive way, which would greatly cause a tremendous positive impact to the durian industry.

## Supplementary Information

Supplementary Figures and Tables.
