# Supplementary material for: Investigation of key chemical species from durian peduncles and their correlations with durian maturity
Source: Sci Rep. 2021 Jun 25;11:13301. doi: 10.1038/s41598-021-92492-6 (PMC8233394; doi:10.1038/s41598-021-92492-6)
Supplement: Supplementary file 1 — Supplementary Figures and Tables. [file 41598_2021_92492_MOESM1_ESM.pdf]

# Investigation of Key Chemical Species from Durian Peduncles and Their Correlations with Durian Maturity

Preeyarad Charoensumran,[a,b] Kornkanya Pratumyot,[c] Tirayut Vilaivan,[a,d] and Thanit Praneenararat[a,b,*]

[a]Department of Chemistry, Faculty of Science, Chulalongkorn University, Phayathai Rd., Pathumwan, Bangkok, 10330, Thailand.

[b]The Chemical Approaches for Food Applications Research Group, Faculty of Science, Chulalongkorn University, Phayathai Rd., Pathumwan, Bangkok, 10330, Thailand.

[c]Organic Synthesis, Electrochemistry & Natural Product Research Unit, Department of Chemistry, Faculty of Science, King Mongkut's University of Technology Thonburi, Bangkok, 10140, Thailand.

[d]Organic Synthesis Research Unit, Department of Chemistry, Faculty of Science, Chulalongkorn University, Phayathai Rd., Pathumwan, Bangkok, 10330, Thailand.

***Corresponding Author**

Email: Thanit.P@chula.ac.th; Tel: +66-2-218-7638

**Table S1.** Conditions for QTRAP-MS analysis of the four marker compounds.

| Compound name | Molecular formular | Monoisotopic mass (Da) | Adduct | Q1 (*m/z*) | Q3 (*m/z*) | CE[(a)] (V) | DP[(b)] (V) | EP[(c)] (V) |
|---|---|---|---|---|---|---|---|---|
| Sucrose | $C_{12}H_{22}O_{11}$ | 342.1162 | $[M+Na]^+$ | 365 | 203 | 40 | 145 | 12.0 |
| Asparagine | $C_4H_8N_2O_3$ | 132.0535 | $[M+H]^+$ | 133 | 74 | 20 | 85 | 6.0 |
| Arginine | $C_6H_{14}N_4O_2$ | 174.1117 | $[M+H]^+$ | 175 | 70 | 30 | 135 | 10.0 |
| Pipecolic acid | $C_6H_{11}NO_2$ | 129.0790 | $[M+H]^+$ | 130 | 84 | 20 | 131 | 10.0 |

(a) CE = Collision Energy
(b) DP = Declustering Potential
(c) EP = Entrance Potential

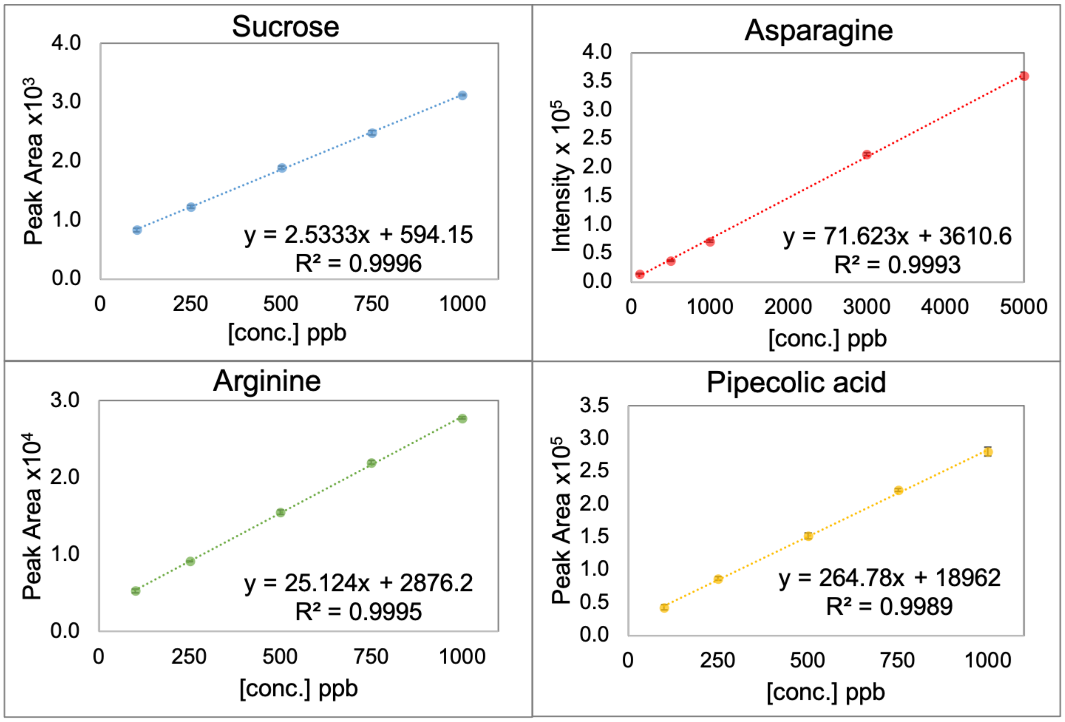


**Figure S1.** Calibration plots of sucrose, arginine, pipecolic acid (0-1000 ppb), and asparagine (0-5000 ppb). All data points were triplicates.

**Table S2.** Numerical data of the sugar amounts (mg/g FW) in the peduncle as shown in Figure 3.

| Sugar | 13 weeks | 15 weeks | 17 weeks |
|---|---|---|---|
| **Fructose** | 0.45 ± 0.30 | 1.54 ± 0.30 | 1.07 ± 0.51 |
| **Glucose** | 0.23 ± 0.10 | 0.94 ± 0.26 | 0.56 ± 0.22 |
| **Sucrose** | 0.62 ± 0.21 | 1.84 ± 0.46 | 2.73 ± 0.55 |

## Sweetness calculations

Sweetness of sucrose was defined to be 1.0,* and those for other compounds were calculated by comparing to sucrose. Since this is related to human perception, the values were reported as ranges, with that of fructose being 0.8 – 1.7, and that of glucose being 0.6 – 0.75. To calculate the sweetness from each sample in this study, the averaged relative values (fructose = 1.25 and glucose = 0.675) were multiplied by sugar concentrations reported in the main article, resulting in the following values in Table S2. It should be noted that this calculation does not take any extra increase of sweetness due to synergistic effect into account.

* Tiefenbacher, K. F. (2017). Chapter Three - Technology of Main Ingredients—Sweeteners and Lipids. In K. F. Tiefenbacher (Ed.), Wafer and Waffle (pp. 123-225): Academic Press, and references therein.

**Table S3.** The relative sweetness of various sugars in durian peduncle.

| Maturity | Fructose | Glucose | Sucrose | Combined sweetness |
|---|---|---|---|---|
| 13 weeks | 0.28 | 0.08 | 0.31 | 0.67 |
| 15 weeks | 0.96 | 0.32 | 0.92 | 2.20 |
| 17 weeks | 0.67 | 0.19 | 1.37 | 2.23 |

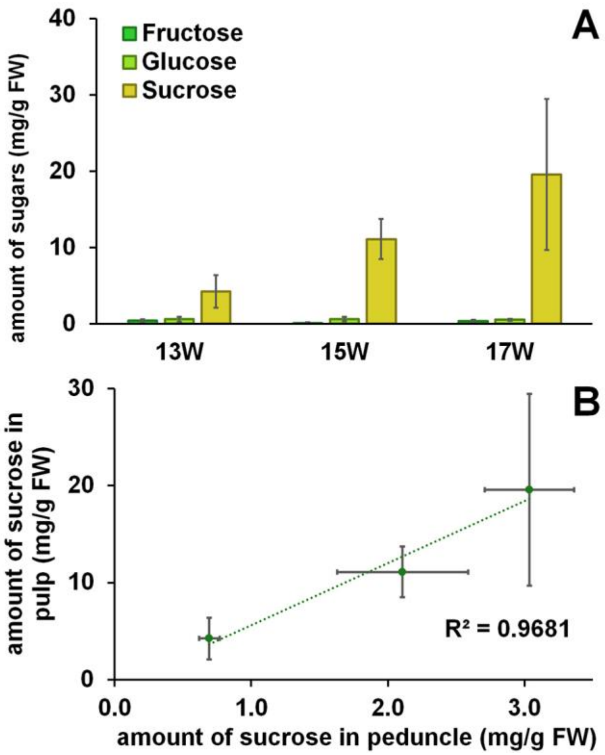


**Figure S2.** (A) The amounts of three sugars in durian pulp at different maturities (13, 15, and 17 weeks) as determined by HPLC-RI. (B) a scattered plot showing the correlation between determined sucrose concentrations (by HPLC-RI) in the pulp and in the peduncle.

**Table S4.** Numerical data of the sugar amounts in the durian pulp (mg/g FW) as shown in Figure S2.

| Sugar | 13 weeks | 15 weeks | 17 weeks |
|---|---|---|---|
| **Fructose** | 0.38 ± 0.17 | 0.08 ± 0.09 | 0.35 ± 0.17 |
| **Glucose** | 0.58 ± 0.31 | 0.60 ± 0.28 | 0.52 ± 0.15 |
| **Sucrose** | 4.25 ± 2.15 | 11.11 ± 2.62 | 19.58 ± 9.89 |

**Table S5.** Numerical data of the amounts of chemical markers as determined by LC-MS/MS (QTRAP) in Figure 6.

| Compound | 13 weeks | 15 weeks | 17 weeks |
|---|---|---|---|
| **Sucrose** | 1.08 ± 0.21 | 2.35 ± 0.12 | 3.03 ± 0.22 |
| **Asparagine** | 0.58 ± 0.15 | 2.59 ± 0.16 | 4.52 ± 0.67 |
| **Arginine** | 0.43 ± 0.11 | 0.55 ± 0.14 | 0.88 ± 0.41 |
| **Pipecolic acid** | 0.06 ± 0.03 | 0.26 ± 0.13 | 0.50 ± 0.29 |
